# The Combination of BH3-Mimetic ABT-737 with the Alkylating Agent Temozolomide Induces Strong Synergistic Killing of Melanoma Cells Independent of p53

**DOI:** 10.1371/journal.pone.0024294

**Published:** 2011-08-29

**Authors:** Steven N. Reuland, Nathaniel B. Goldstein, Katie A. Partyka, David A. Cooper, Mayumi Fujita, David A. Norris, Yiqun G. Shellman

**Affiliations:** 1 University of Colorado Denver, School of Medicine, Department of Dermatology, Aurora, Colorado, United States of America; 2 Department of Veterans Affairs Medical Center, Dermatology Section, Denver, Colorado, United States of America; Bauer Research Foundation, United States of America

## Abstract

Metastatic melanoma has poor prognosis and is refractory to most conventional chemotherapies. The alkylating agent temozolomide (TMZ) is commonly used in treating melanoma but has a disappointing response rate. Agents that can act cooperatively with TMZ and improve its efficacy are thus highly sought after. The BH3 mimetic ABT-737, which can induce apoptosis by targeting pro-survival Bcl-2 family members, has been found to enhance the efficacy of many conventional chemotherapeutic agents in multiple cancers. We found that combining TMZ and ABT-737 induced strong synergistic apoptosis in multiple human melanoma cell lines. When the drugs were used in combination in a mouse xenograft model, they drastically reduced tumor growth at concentrations where each individual drug had no significant effect. We found that TMZ treatment elevated p53 levels, and that the pro-apoptotic protein Noxa was elevated in TMZ/ABT-737 treated cells. Experiments with shRNA demonstrated that the synergistic effect of TMZ and ABT-737 was largely dependent on Noxa. Experiments with nutlin-3, a p53 inducer, demonstrated that p53 induction was sufficient for synergistic cell death with ABT-737 in a Noxa-dependent fashion. However, p53 was not necessary for TMZ/ABT-737 synergy as demonstrated by a p53-null line, indicating that TMZ and ABT-737 together induce Noxa in a p53-independent fashion. These results demonstrate that targeting anti-apoptotic Bcl-2 members is a promising method for treating metastatic melanoma, and that clinical trials with TMZ and Bcl-2 inhibitors are warranted.

## Introduction

The incidence of metastatic melanoma has increased rapidly in recent decades, but unfortunately there has been little improvement in therapeutic efficacy [1]. Dacarbazine is the standard first-line treatment for advanced melanoma, but its response rate is poor, averaging around 15% with no improvement in survival duration [1], [2]. Temozolomide (TMZ), which spontaneously decomposes into the active metabolite of dacarbazine [3], is frequently used “off-label” in place of dacarbazine because of its ease of use and bioavailability; however, its response rate is equally poor. Chemotherapeutic agents that can be combined with TMZ to increase its response rate are therefore highly sought after, as an effective combination would have immediate clinical application. Agents that have been combined with TMZ in clinical trials include arsenic trioxide and ascorbic acid [4], cisplatin [5], and thalidomide [6]; unfortunately, they were found to have no benefit.

Whether and to what degree TMZ induces apoptosis in melanoma cells is a subject of debate. Some studies have shown that clinically relevant doses of TMZ do not induce significant levels of apoptosis in melanoma cells *in vitro*, potentially explaining its poor clinical response rate [7], [8]. However, recently, Naumann and coworkers found that apoptosis is the major mode of death for melanoma cells exposed to TMZ, but that it requires time for double-stranded DNA breaks to occur, and thus apoptosis is not observed with short treatment times (72 h or less) [9]. Enhancing the apoptotic potential of TMZ, for example by triggering apoptosis under situations in which cell cycle arrest would predominate, is a promising means of enhancing the efficacy of TMZ in melanoma patients.

Many cancers, particularly melanomas, are resistant to apoptosis by upregulation of anti-apoptotic Bcl-2 family members. As a means of overcoming this resistance, agents that mimic pro-apoptotic BH3-only proteins, such as ABT-737, have been developed. ABT-737 potently inhibits anti-apoptotic Bcl-2 family members Bcl-2, Bcl-X_L_, and Bcl-W, and as such effectively mimics the BH3-only protein Bad [10]. It has been shown to enhance the activity of a variety of cytotoxic drugs, and is thus an excellent candidate for rational drug combinations [11]. We previously found that resistance to ABT-737 in melanoma cells is mediated by the anti-apoptotic Bcl-2 member Mcl-1, which ABT-737 does not inhibit [12]. The induction of Noxa, which selectively inhibits Mcl-1, via the protease inhibitors MG-132 [12] or Bortezomib (unpublished data) is able to overcome this resistance. Knockdown of Mcl-1 results in increased sensitivity to ABT-737, while knockdown of Noxa protects cells from the killing induced by the combination of the two drugs [12]. Numerous additional *in vitro* studies for both melanoma and other cancer cells have shown that the Mcl-1/Noxa ratio is critical for determining resistance or sensitivity to ABT-737 [13], [14], [15], [16], [17], [18].

In the present study, we tested the BH3-only mimetic ABT-737 in combination with the commonly used alkylating agent TMZ, and found strong synergistic induction of apoptosis in several melanoma cell lines within a short time period, and a significant reduction in tumor growth in a mouse xenograft model. We found that Noxa was induced by the combination treatment, but not by single drug treatments, and that knockdown of Noxa almost completely abrogated cell death induced by the combination. Although induction of p53 was sufficient to cause Noxa-mediated cell death, it was not necessary, indicating that the ABT-737/TMZ combination induces Noxa through a p53-independent pathway.

## Results

### ABT-737 synergistically induces apoptosis in melanoma cells when combined with temozolomide

**Figure 1:**
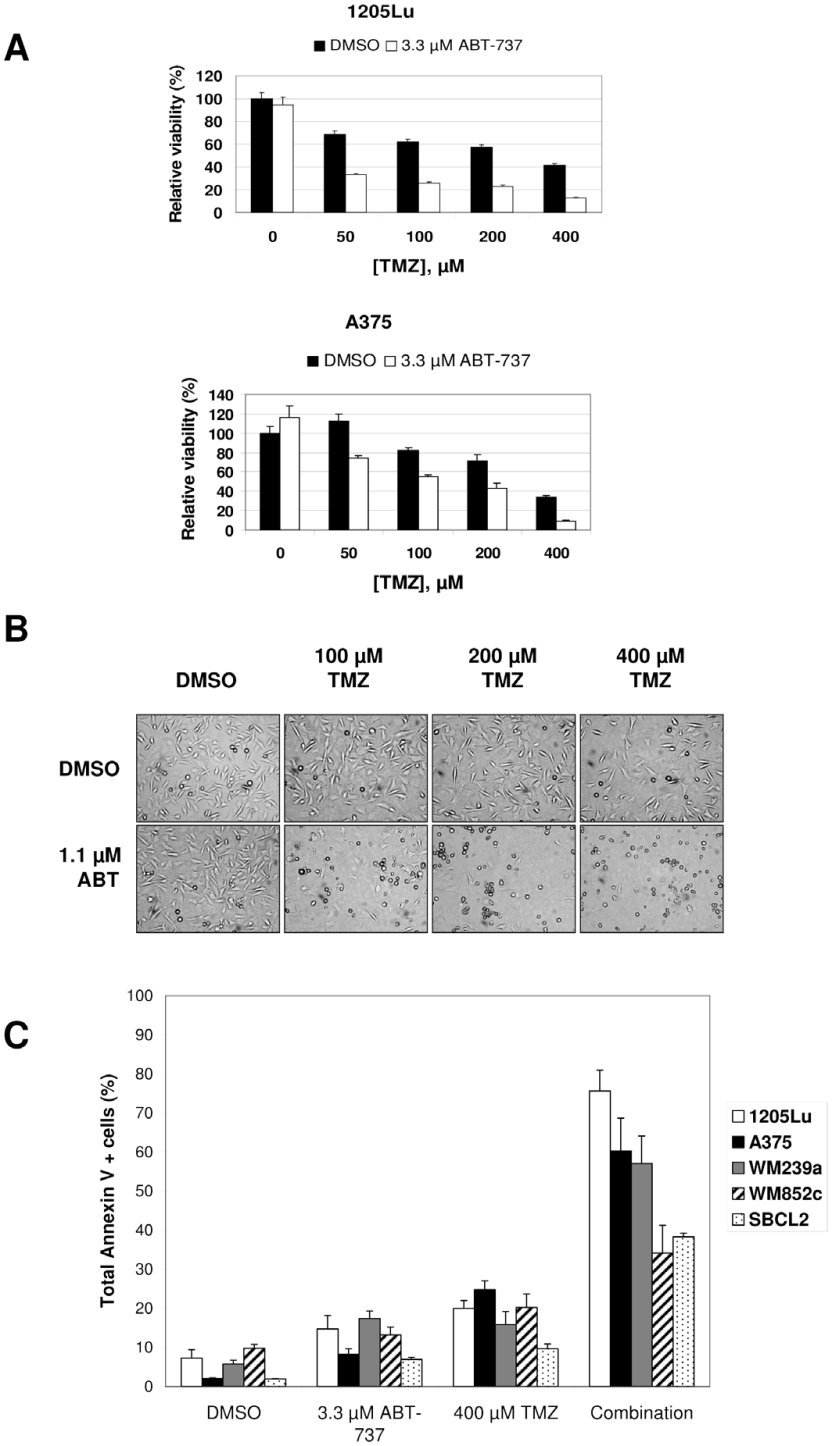
TMZ and ABT-737 cause strong, synergistic cell death in melanoma cell lines. (**A**) MTS assays of 1205Lu (top) and A375 (bottom) cell lines treated with varying doses of TMZ with or without 3.3 µM ABT-737 for 96 h. (**B**) The visual appearance of cells (100× magnification) treated with varying doses of TMZ with or without 1.1 µM ABT-737 for 72 h. TMZ treatment primarily reduced cell proliferation, while the combination treatment caused cell death. (**C**) Annexin V assays for various cell lines treated with TMZ (400 µM) and ABT-737 (3.3 µM) for 72 h.

MTS experiments (Fig. 1A and data not shown) showed that TMZ alone reduced total viability, and that this was reduced further in the presence of ABT-737. IC_50_ values for each drug at 72 h are listed in Table S1, and time-course data are shown in Fig. S1. Median effect analysis showed that the combination was synergistic over a wide range of drug concentrations at 72 h (Fig. S2), with combination index (CI) values ranging from 0.1 to 0.4 for 1205Lu and 0.3 to 0.8 for A375. The visual appearance of the cells (Fig. 1B) made it clear that the combination of ABT-737 and TMZ induced cell death whereas TMZ alone primarily reduced cell proliferation by 72 h. To quantify the level of apoptosis in combination treatments compared to single agent treatments, we performed Annexin V assays after cells were exposed to 400 µM TMZ alone, 3.3 µM ABT-737 alone, or both agents in combination for 72 h for several cell lines. Figure 1C shows that ABT-737 and TMZ alone induced little cell death above the controls treated with vehicle. For combination treatments however, high levels of cell death were found for all cell lines, indicating a synergistic effect between TMZ and ABT-737.

### TMZ and ABT-737 combination treatment induces Noxa and p53 expression

**Figure 2:**
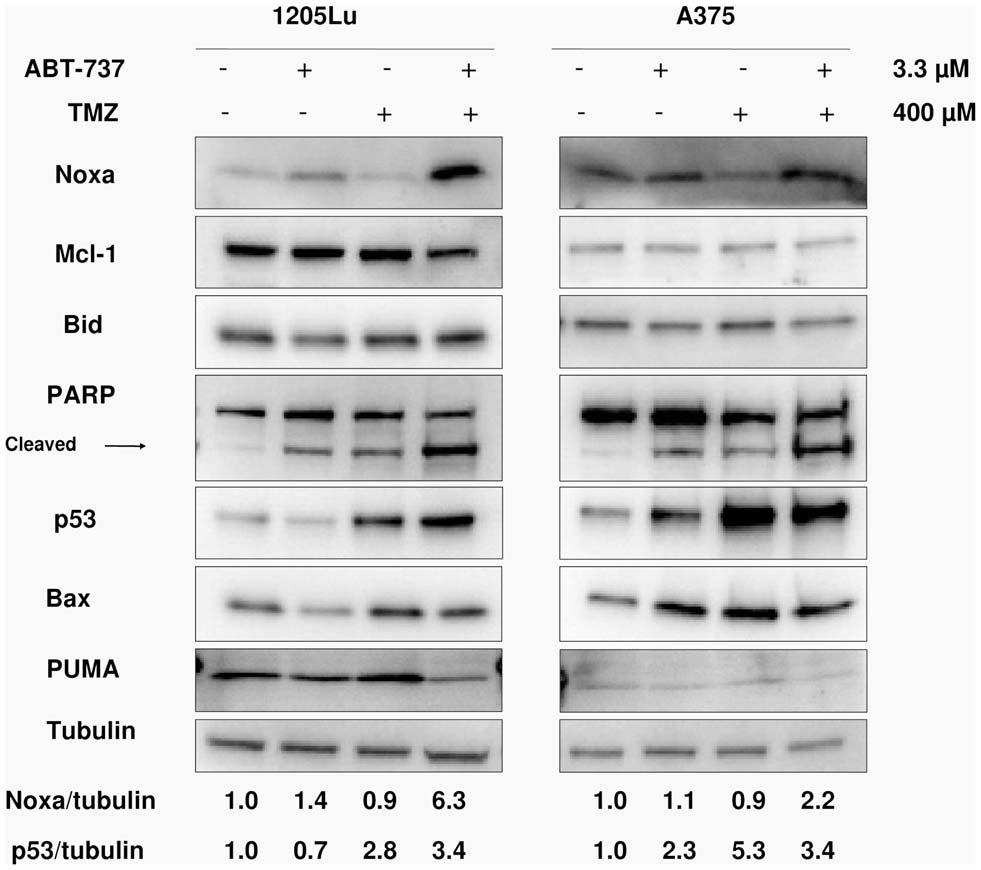
p53 and Noxa are increased in TMZ/ABT-737 treated cells. Immunoblots are of lysates from Annexin V experiments shown in Fig. 1C with 72 h drug exposure. PARP cleavage indicates high levels of apoptosis in combination treated cells. TMZ treatment alone increases levels of p53, but only the combination treatment increases Noxa. Other Bcl-2 family members tested remained unchanged. Relative ratios of Noxa and p53 to tubulin are shown below, with each value normalized to the control.

Western blotting (Fig. 2) for 1205Lu and A375 demonstrated elevated levels of Noxa (roughly 6- and 2-fold, respectively) in cells treated with a combination of TMZ and ABT-737, though Noxa levels in cells treated only with TMZ remained unchanged. Mcl-1 levels were unchanged, resulting in a highly increased Noxa/Mcl-1 ratio in the combination group. Additionally, TMZ treatment greatly increased the levels of p53 (roughly 3- and 5-fold for 1205Lu and A375, respectively), which is known to induce pro-apoptotic Bcl-2 family members. However, levels of PUMA, Bid, and Bax were unchanged, and there was no indication of Bid truncation (tBid). These results imply that the synergy of TMZ and ABT-737 may be mediated through either increased Noxa or p53 levels, but that other pro-apoptotic Bcl-2 family members tested are unlikely to play a role.

### ABT-737 synergy with TMZ is Noxa-dependent

**Figure 3:**
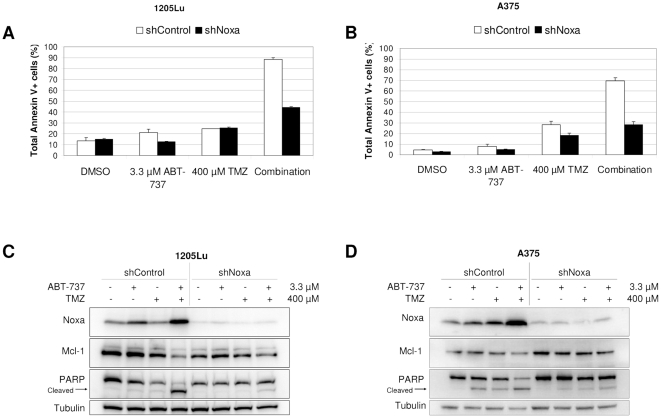
Synergy between TMZ and ABT-737 is Noxa-dependent. Annexin V assays of 1205Lu (**A**) and A375 (**B**) shNoxa and shControl cells treated with or without 400 µM TMZ and 3.3 µM ABT-737 for 72 h. Immunoblots of cell lysates from Annexin V experiments for 1205Lu (**C**) and A375 (**D**) cells show Noxa knockdown and lack of PARP cleavage for shNoxa lines, while high levels of Noxa exist in combination-treated shControl lines.

To test the involvement of Noxa in TMZ and ABT-737 synergy, we created 1205Lu and A375 cell lines stably expressing short hairpin RNA (shRNA) against Noxa (shNoxa). Western blotting confirmed the knockdown of Noxa in these lines (Fig. 3C, D). Annexin V experiments (Fig. 3A, B) showed that the synergistic killing effect of TMZ and ABT-737 was almost completely abrogated in shNoxa lines, indicating that Noxa is necessary for the synergy. Western blots (Fig. 3C, D) show greatly reduced PARP cleavage in shNoxa cells compared to shControl cells, corroborating the Annexin V assays and showing that Noxa is necessary for apoptosis in the combination treatment.

### Induction of p53 is sufficient, but not necessary, to induce synergistic cell death with ABT-737 in melanoma cell lines

**Figure 4:**
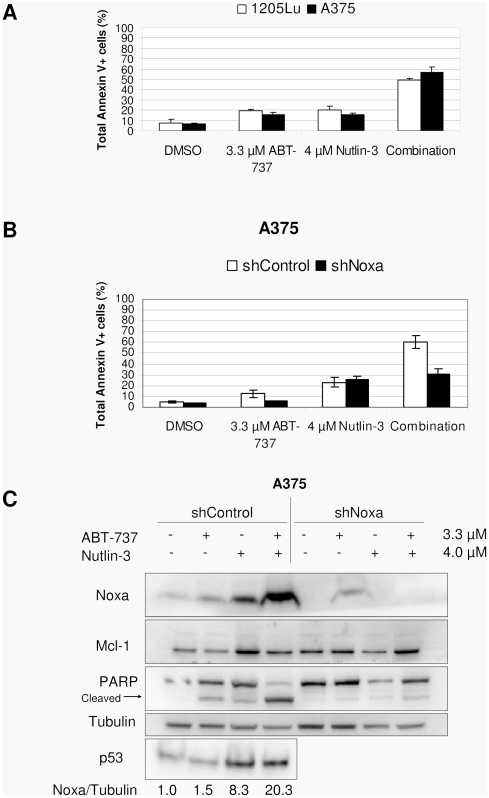
p53 induction through nutlin-3 causes synergy with ABT-737 in a Noxa-dependent fashion. (**A**) Annexin V assays of 1205Lu and A375 cells treated with or without 4 µM nutlin-3 and 3.3 µM ABT-737. (**B**) A375 shNoxa and shControl cells treated with or without 4 µM nutlin-3 and 3.3 µM ABT-737. (**C**) Immunoblots of lysates from the Annexin V experiments shown in B show Noxa knockdown and lack of PARP cleavage in the shNoxa line. Relative ratios of Noxa to tubulin are shown below, with each value normalized to the control.

To test if p53 induction alone was sufficient to cause synergistic apoptosis with ABT-737, we tested the compound nutlin-3, which increases p53 levels by inhibiting p53 degradation through MDM2-mediated ubiquitination of p53 [19]. We performed Annexin V assays for A375 and 1205Lu cells treated with the drugs for 72 h. Fig. 4A shows that nutlin-3 and ABT-737 induced little apoptosis at 4 µM and 3.3 µM, respectively, but both drugs together induced apoptosis in roughly half of the cells. The visual appearance of cells (not shown) indicated that nutlin-3 alone mostly reduced cell proliferation, whereas cell death was most common when combined with ABT-737, a phenomenon highly similar to TMZ. Immunoblots of lysates from the Annexin experiments (Fig. 4C) show that Noxa was increased in nutlin-3-treated cells (roughly 8-fold), but increased more so in the combination treatments (roughly 20-fold). When shNoxa lines were treated with the combination of nutlin-3 and ABT-737 (Fig. 4B), apoptosis was almost completely abrogated, similar to TMZ combination treatments.

**Figure 5:**
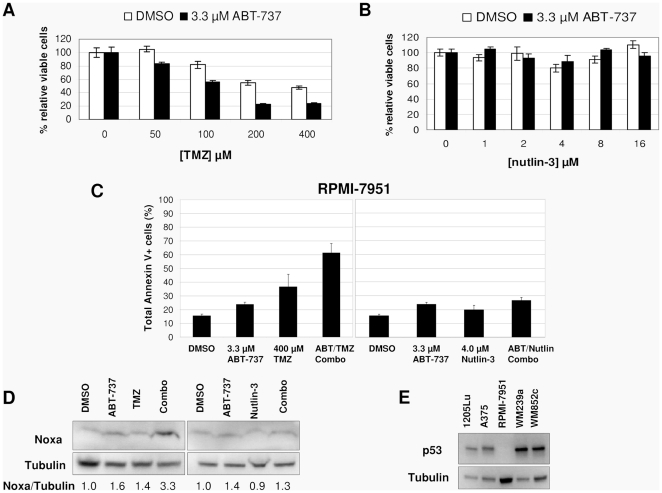
p53 is not necessary for synergy between TMZ and ABT-737. MTS assays of RPMI-7951 (a p53-null line) with varying doses of TMZ (**A**) or nutlin-3 (**B**) with or without 3.3 µM ABT-737. (**C**) Annexin V assays of RPMI-7951 cells treated with or without 400 µM TMZ and 3.3 µM ABT-737 (left) or 4 µM nutlin-3 and 3.3 µM ABT-737 (right). (**D**) Immunoblots of cell lysates from the Annexin V experiments shown in C show Noxa induction in TMZ/ABT-737-treated cells but not in nutlin-3/ABT-737-treated cells. Relative ratios of Noxa to tubulin are shown below, with each value normalized to the control. (**E**) Immunoblots of several melanoma cell lines show that p53 is undetectable in RPMI-7951 cells.

To further test whether p53 induction is necessary for the synergistic effect of TMZ and ABT-737, we used the cell line RPMI-7951, which is homozygous for a p53 nonsense mutation at S166 [20]. MTS and Annexin V assays (Fig. 5B, C) showed that RPMI-7951 cells were unaffected by nutlin-3 as expected. However, surprisingly, RPMI-7951 cells underwent synergistic cell death when treated with TMZ and ABT-737 (Fig. 5A, C). Noxa was induced in TMZ/ABT-737 combination treatments by over 3-fold compared to the control (Fig. 5D), similar to p53 wild-type cell lines. We were unable to detect any p53 in RPMI-7951 cells (Fig. 5E), even when treated with TMZ or nutlin-3, indicating that the p53 message was either degraded through nonsense-mediated mRNA decay, or that the truncated protein was quickly degraded. We therefore regard this cell line as p53-null. In spite of this, we found that it was sensitive to TMZ and ABT-737 combination treatments. These results demonstrate that induction of Noxa is necessary to cause synergy with ABT-737, and that while induction of p53 is sufficient for synergistic cell death (mediated through Noxa), it is not necessary.

### TMZ and ABT-737 reduce tumor growth in a mouse xenograft model

**Figure 6:**
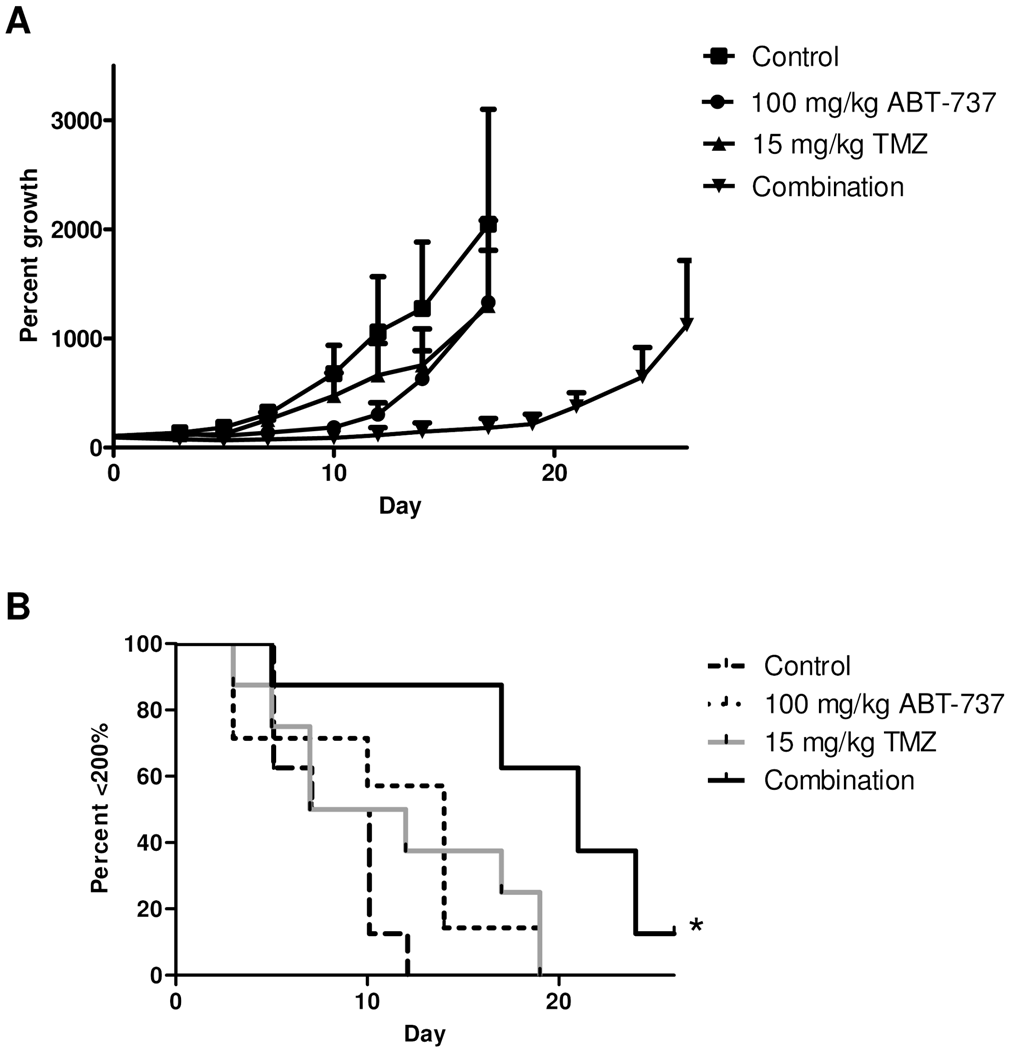
TMZ and ABT-737 reduce tumor growth in a mouse xenograft model. (**A**) Tumor growth curves. (**B**) Kaplan-Meier curves for tumor doubling times. * p = 0.0005 vs. control.

We administered ABT-737, TMZ, or both drugs together in a mouse xenograft model with A375 cells injected subcutaneously into each flank. As shown in Fig. 6, ABT-737 and TMZ treatments alone had little effect on tumor growth compared to the control (p = 0.2570 and 0.3252, respectively). However, the combination treatment highly significantly slowed the rate of tumor growth compared to the control (p = 0.0005) and to the individual drug treatment groups (p = 0.0037 and 0.0276 vs. ABT-737 and TMZ, respectively). Notably, the mice that received the drug combination appeared normal without weight loss (data not shown) or any other noticeable side effects. These results imply that the combination of ABT-737 and TMZ can reduce the growth of melanoma tumors *in vivo*, and that the drug combination is likely to be safe.

## Discussion

Metastatic melanoma is responsible for roughly 80% of all skin cancer deaths and has a 5-year survival rate of only 14%, even with TMZ as a standard treatment [21]. Thus, new treatment options that can be quickly moved to clinic are desperately needed. Melanomas are notoriously resistant to apoptosis induction by TMZ and other chemotherapeutic agents [22]. Although much attention has been focused on DNA damage repair mechanisms in mediating this resistance [23], targeting apoptotic pathways directly is a potential means of improving the poor clinical efficacy of TMZ.

To enhance the ability of TMZ to induce apoptosis, we combined it with the BH3-only mimetic ABT-737. Our results with TMZ alone are consistent with those of previous studies showing that TMZ primarily reduces the rate of cell growth but induces little apoptosis within short time frames, even though we used a relatively high dose [7], [8], [9]. However, when used in combination with ABT-737, TMZ strongly induced apoptosis in multiple cell lines by 72 h, in some cases resulting in nearly complete cell death. We also found that the combination was effective in melanoma cells carrying either BRAF or NRAS activating mutations, since A375, 1205Lu, and WM239a lines each have *BRAF^V600^* mutations, while WM852c and SBCL2 lines have NRAS*^Q61^* mutations. We also found two cell lines, SK-Mel 28 and 451Lu, with high levels of methylguanine methyltransferase (MGMT), an enzyme that directly repairs methylguanine adducts and is implicated in TMZ resistance [24]. These lines were completely resistant to the effects of TMZ, and hence also resistant to the drug combination (data not shown). However, the addition of an MGMT inhibitor, O^6^-benzylguanine, restored TMZ sensitivity and the synergistic effect of the TMZ/ABT-737 drug combination (data not shown), indicating that MGMT does not affect the mechanism of drug synergy.

We found strong induction of p53 with TMZ treatment, and experiments with the p53-inducing agent nutlin-3 support the idea that induction of p53 can cause synergistic killing when combined with ABT-737 in some cell lines. The effects of nutlin-3 on 1205Lu and A375 melanoma cell lines, both alone and in combination with ABT-737, were remarkably similar to those of TMZ. This indicates that combining ABT-737 with agents that induce p53 is also a promising strategy for treating p53 wild-type melanomas. However, we found that RPMI-7951, a p53-null line, was sensitive to TMZ/ABT-737 but not to nutlin-3/ABT-737, demonstrating that p53 is not necessary for synergistic cell death induced by TMZ and ABT-737. This result is consistent with previous studies showing that p53 status is irrelevant to the effect of TMZ in melanoma cells [9]. It also implies that combining ABT-737 with TMZ may be a better strategy than inducing p53, since the former should work regardless of p53 status.

The apoptotic pathway is regulated by Bcl-2 family members, and high levels of the anti-apoptotic Bcl-2 members are implicated in the resistance of cancer cells to apoptosis. ABT-737 promotes apoptosis by serving as a BH3-only mimetic, counteracting the increased levels of anti-apoptotic Bcl-2 members. Our previous work has shown that melanoma cells are only slightly sensitive to ABT-737 at high doses, and that this resistance is mediated exclusively through Mcl-1 [12]. The inhibition of Mcl-1, either directly or through the induction of proteins antagonizing Mcl-1, should therefore greatly increase the cell killing ability of ABT-737. Noxa, BID (in its truncated form, tBID), and PUMA are known to sequester Mcl-1 and serve as pro-apoptotic molecules [25]. BAX is a main mediator of mitochrondrial apoptosis, so increased levels of BAX can potentially overcome sequestration by Mcl-1. However, we found that TMZ alone failed to induce any of these proteins significantly and consistently above levels seen in multiple vehicle-treated cell lines. In contrast, in TMZ/ABT-737 combination-treated cells, there was a notable increase of Noxa in multiple cell lines.

Experiments with shRNA against Noxa demonstrated that the synergistic killing of TMZ and ABT-737 is mediated at least in part through Noxa. Identical experiments using nutlin-3 in lieu of TMZ showed that Noxa is increased by nutlin-3 and especially in the combination treatments, and that Noxa is also necessary for nutlin-3/ABT-737-mediated cell death in A375 cells, indicating that Noxa is also the key downstream target of p53 induced by nutlin-3. Additionally, we tested A375 cells in which BIM and PUMA were knocked-down by shRNA. MTS assays using these cells treated with TMZ/ABT-737 indicated that synergistic cell death is not mediated through these proteins (data not shown). We therefore concluded that Noxa is the main mediator of cell death induced by TMZ/ABT-737.

We also found that Noxa was increased upon TMZ/ABT-737 treatment in both the p53-null cells and wild-type p53 cells. The combination of TMZ and ABT-737 must therefore cause p53-independent Noxa induction. However, nutlin-3 treatment increased Noxa only in wild-type p53 cells. These results suggest that TMZ and nutlin-3 are inducing Noxa through different mechanisms. Nutlin-3 alone induced Noxa, providing a simple explanation for how it synergizes with ABT-737, but surprisingly, TMZ alone did not. Instead, TMZ only induced Noxa when combined with ABT-737, and did so even in the complete absence of p53. So while nutlin-3 appears to be inducing Noxa through a p53-dependent mechanism, TMZ must be doing so through a p53-independent mechanism, one that operates only in the presence of ABT-737. It is unclear at present what this mechanism is, or why p53 induced by TMZ is insufficient to induce Noxa by itself. Although there are many reports of p53-independent Noxa induction, the mechanisms responsible are often undetermined. It is known that Noxa can be induced via cMyc during proteasome inhibition [26], and that E2F1 can induce Noxa along with other BH3-only proteins [27]. However, we did not find elevated cMyc levels in any of our treatment groups, and paradoxically, E2F1 levels were significantly decreased in TMZ and TMZ/ABT-737 treated cells (data not shown). The mechanism by which TMZ/ABT-737 induces Noxa will require further study.

Results from our *in vivo* mouse model are consistent with our *in vitro* data. The combination of TMZ and ABT-737 caused tumors to grow at a significantly lower rate compared to either control mice or to mice treated with either drug alone. The drugs used individually were at doses where neither had a significant effect compared to the control. Since the dose used for TMZ was relatively low (15 mg/kg), these results imply that when combined with ABT-737, clinically relevant doses of TMZ may cause tumor shrinkage in human patients, not merely a reduction of growth. Notably, there were no readily apparent adverse side effects such weight loss, lethargy, or flaky skin as seen with Bortezomib treatment (unpublished observation), indicating that the drug combination is likely to be minimally toxic.

Our *in vivo* and *in vitro* data, plus the apparent safety of the drug combination, imply that a combination of TMZ and ABT-737 (or its oral form, ABT-263) is warranted for clinical trial. Melanoma is an especially good candidate for treatment with this drug combination, because TMZ is already in widespread use for treating metastatic melanoma, providing an avenue for immediate clinical benefit. Additionally, while melanomas have a surprisingly low rate of p53 mutations (<10%) [28], our data indicate that the drug combination should work irrespective of p53 status.

In conclusion, we found that TMZ and ABT-737 synergize to promote cell death in multiple melanoma cell lines, and dramatically reduce tumor growth in a mouse model even when used at low doses. Our data indicate that this synergy occurs through the p53-independent induction of Noxa, although p53-dependent induction of Noxa through nutlin-3 produces a similar effect and may be effective against p53 wild-type tumors. Our results show that combining TMZ with anti-apoptotic Bcl-2 inhibitors is a promising method for treating melanomas, and that clinical trials with TMZ and ABT-737-like compounds are warranted.

## Materials and Methods

### Ethics statement

This study was carried out in strict accordance with the recommendations in the Guide for the Care and Use of Laboratory Animals of the National Institutes of Health. All experiments were approved by the Institutional Animal Care and Use Committee of the University of Colorado Denver (protocol number 88509(09)1E).

### Reagents

Temozolomide (99% purity), nutlin-3, and O^6^-benzylguanine were obtained from Sigma-Aldrich (St. Louis, MO). ABT-737 was kindly provided by Abbott Laboratories (Abbott Park, IL).

### Cell lines and culture conditions

The following human melanoma cell lines were used: metastatic cell lines A375, 1205Lu, SK-Mel 28, 451Lu, and RPMI-7951 were obtained from ATCC (Manassas, VA). Metastatic cell lines WM852c and WM239a, and radial growth phase cell line SBCL2, were kindly provided by Dr. Meenhard Herlyn (Wistar Institute, Philadelphia, PA). Cells were maintained in RPMI1640 media (Invitrogen, Grand Island, NY) with 10% fetal bovine serum (Gemini Bio-Products, Inc., West Sacramento, CA). A375, 1205Lu, SK-Mel 28, 451Lu, and RPMI-7951 lines each have *BRAF^V600E^* mutations. WM239a has *BRAF^V600D^*, WM852c has *NRAS^Q61R^*, and SBCL2 has the *NRAS^Q61K^* mutation. Cell lines with a BRAF mutation do not have common mutations in NRAS (exons 1 or 2), and lines with NRAS mutations do not have BRAF mutations (exons 11 or 15). RPMI-7951 is homozygous for a nonsense mutation in the p53 gene at codon 166 [20]. All other cell lines used in Annexin V assays are p53 wild-type. Cell lines SK-Mel 28 and 451Lu have high levels of methylguanine methyltransferase (MGMT), while A375, WM852c, and SCBL2 have 2-3 fold lower levels. We could not detect MGMT in 1205Lu or WM239a lines. None of the cell lines used in this study have been previously reported to be deficient in mismatch repair.

### Measurement of cell proliferation and apoptosis

The Cell Titer 96TM Aqueous One solution cell proliferation assay (MTS assay; Promega Corp., Madison, WI) was used to quantify cell viability. Assays were performed according to the manufacturer's instructions. Control experiments in which TMZ and ABT-737 were added to wells without cells demonstrated that the drugs do not react with the MTS reagent or otherwise interfere with the assay. The Annexin V-FITC Apoptosis Detection Kit (BD Biosciences, San Jose, CA) was used to quantify apoptosis according to the manufacturer's protocol. Cells were analyzed by flow cytometry using a Beckman Coulter FC500 with CXP software (Hialeah, FL) in the University of Colorado Cancer Center Flow Cytometry Core.

### Creation of short hairpin RNA transduced cell lines

Short hairpin RNA (shRNA) expressing lines against various Bcl-2 family members, or scrambled control, were constructed using shRNA Lentiviral Particles from Santa Cruz Biotechnology (Santa Cruz, CA) according to the manufacturer's instructions with slight modification. Briefly, cells were seeded in 12-well plates for 24 h at concentrations sufficient to reach 50% confluency. The media was removed from each well and replaced with 1 ml of chilled polybrene working solution (5 µg/ml in RPMI1640 medium) and incubated at RT for 5 min. The solution was removed and replaced with 1 ml of chilled polybrene working solution with up to 20 µl of viral particle and incubated at 37°C. The solution was then removed 24 h later, cells were rinsed once with fresh media, and then 1 ml of fresh media was added. Cells were grown until sufficient numbers were available for selection. Transduced cells were selected by supplementing the media with puromycin (1–4 µg/ml) for several days, replacing the medium with fresh puromycin-containing medium every 3–4 days until resistant colonies could be identified. Knockdown of genes of interest was measured by immunoblotting of cell lysates.

### Immunoblot

Cells, both floating and adherent, were harvested with 1x Laemmli Sample Buffer (Bio-Rad, Hercules, CA). Samples were used in the standard western blot analysis protocol as described previously [29]. Blots were developed with HRP substrate (SuperSignal West Pico or Femto solutions, Pierce, Rockford, IL) for 5 min at room temperature, and analyzed using a Chemi-doc chemiluminescence detector (Bio-Rad, Hercules, CA). The following antibodies were used at suggested dilutions from the manufacturers: Bax, PARP, MGMT, and α/β Tubulin were from Cell Signaling Technology (Danvers, MA); Noxa was from EMD Biosciences, Inc. (San Diego, CA); Mcl-1 was from BD Biosciences (San Jose, CA); Bcl-2 was from Dako (Glostrup, Denmark); p53 was from Santa Cruz Biotechnology; PUMA was from Sigma; and HRP-conjugated goat anti-mouse and anti-rabbit antibodies were from Jackson Immuno-Research (West Grove, PA). Immunoblots were typically performed 2–3 times for multiple cell lines, and representative examples are shown. Immunoblot data was quantified using Quantity One software (Bio-Rad).

### Mouse xenograft model

Female NCRNU nude mice, aged 5–6 weeks, were purchased from Taconic (Hudson, NY). Each mouse was subcutaneously injected on each flank with 2 million A375 cells in a 100 µl volume consisting of 50% BD Matrigel Matrix (BD Biosciences) prepared according to the manufacturer's protocol. Drug treatments began after tumors reached approximately 100 mm^3^, at around 1 week. Mice were randomly divided into four treatment groups consisting of 10 tumors each group: 1) vehicle only, 2) ABT-737 only, 3) TMZ only, 4) TMZ plus ABT-737. TMZ and ABT-737 were administered at 15 mg/kg and 100 mg/kg, respectively. All mice received either the drug or vehicle for both drugs. ABT-737 was prepared fresh every 7 days by dissolving it in vehicle consisting of 65% D5W, 30% propylene glycol, 5% Tween-80, pH 1.0. The pH was raised to ∼3.5 after the drug was fully dissolved. ABT-737 or vehicle was administered daily for 21 days via intraperitoneal (i.p.) injection. A stock solution of TMZ dissolved in DMSO was diluted in normal saline (0.9%) each day prior to use, and TMZ or normal saline vehicle was administered on days 1–5 and 11–15 via i.p. injection. On days when both drugs were administered, TMZ was administered at least 5 h prior to ABT-737.

### Statistical analysis

The anti-proliferative effects from MTS assays were entered into Calcusyn software (Biosoft, Ferguson, MO) to calculate IC_50_ values for individual drugs and to determine whether the treatment combinations had synergistic, additive, or antagonistic effects using the Chou-Talalay method [30]. When the CI value is less than 1, it indicates synergistic effects, and the lower the CI, the stronger the synergism. For animal experiments, Kaplan-Meier curve analysis was used to compare individual tumor doubling rates. Log-rank (Mantel-Cox) tests were used to compare Kaplan-Meier curves with the program GraphPad Prism 5 (GraphPad Software, San Diego, CA), and *p*-values of 0.05 and below were considered significant.

## Supporting Information

Table S1 **IC50 values (µM) for two melanoma cell lines for two drugs with 72 h treatment times.** Values in parentheses represent the 95% confidence interval.
(PDF)

Figure S1 **Time-course experiments for cells treated with TMZ with or without ABT-737.** MTS assays were performed for each 24 h time-point between 48 and 120 h for 1205Lu (**A**, **B**) and A375 (**C**, **D**) cell lines. Experiments included varying concentrations (µM) of TMZ alone (**A**, **C**) or varying concentrations of TMZ with 3.3 µM of ABT-737 (**B**, **D**). Results for each cell line are normalized to DMSO controls (0 TMZ) set at 100% for each time-point, and error bars represent +/- SE of 3 measurements.
(PDF)

Figure S2 **Fraction affected vs. Combination Index (CI) for melanoma cells treated with TMZ and ABT-737.** 1205Lu cells (**A**) or A375 cells (**B**) were treated with escalating doses of TMZ (50, 100, 200, 400 µM) and ABT-737 (0.94, 1.88, 3.75, 7.5 µM) for 72 h and then subjected to MTS assays. CI plots were generated using Calcusyn software according to the Chou-Talalay method, and algebraic simulations are shown +/- estimated s.d.
(PDF)
