# Supplementary material for: The Combination of BH3-Mimetic ABT-737 with the Alkylating Agent Temozolomide Induces Strong Synergistic Killing of Melanoma Cells Independent of p53
Source: PLoS One. 2011 Aug 29;6(8):e24294. doi: 10.1371/journal.pone.0024294 (PMC3163662; doi:10.1371/journal.pone.0024294)
Supplement: Table S1 — IC50 values (µM) for two melanoma cell lines for two drugs with 72 h treatment times. Values in parentheses represent the 95% confidence interval. (PDF) [file pone.0024294.s001.pdf]

Supporting Information Table 1. IC_{50} values (μM) for two melanoma cell lines for two drugs with 72 h treatment times. Values in parentheses represent the 95% confidence interval.

	**ABT-737**	**TMZ**
**A375**	16.1 (6.09-42.5)	401 (216-746)
**1205Lu**	27.1 (6.58-58.1)	626 (351-1484)
