# Supplementary material for: The Combination of BH3-Mimetic ABT-737 with the Alkylating Agent Temozolomide Induces Strong Synergistic Killing of Melanoma Cells Independent of p53
Source: PLoS One. 2011 Aug 29;6(8):e24294. doi: 10.1371/journal.pone.0024294 (PMC3163662; doi:10.1371/journal.pone.0024294)
Supplement: Figure S1 — Time-course experiments for cells treated with TMZ with or without ABT-737. MTS assays were performed for each 24 h time-point between 48 and 120 h for 1205Lu (A, B) and A375 (C, D) cell lines. Experiments included varying concentrations (µM) of TMZ alone (A, C) or varying concentrations of TMZ with 3.3 µM of ABT-737 (B, D). Results for each cell line are normalized to DMSO controls (0 TMZ) set at 100% for each time-point, and error bars represent +/- SE of 3 measurements. (PDF) [file pone.0024294.s002.pdf]

Supporting Information Figure 1

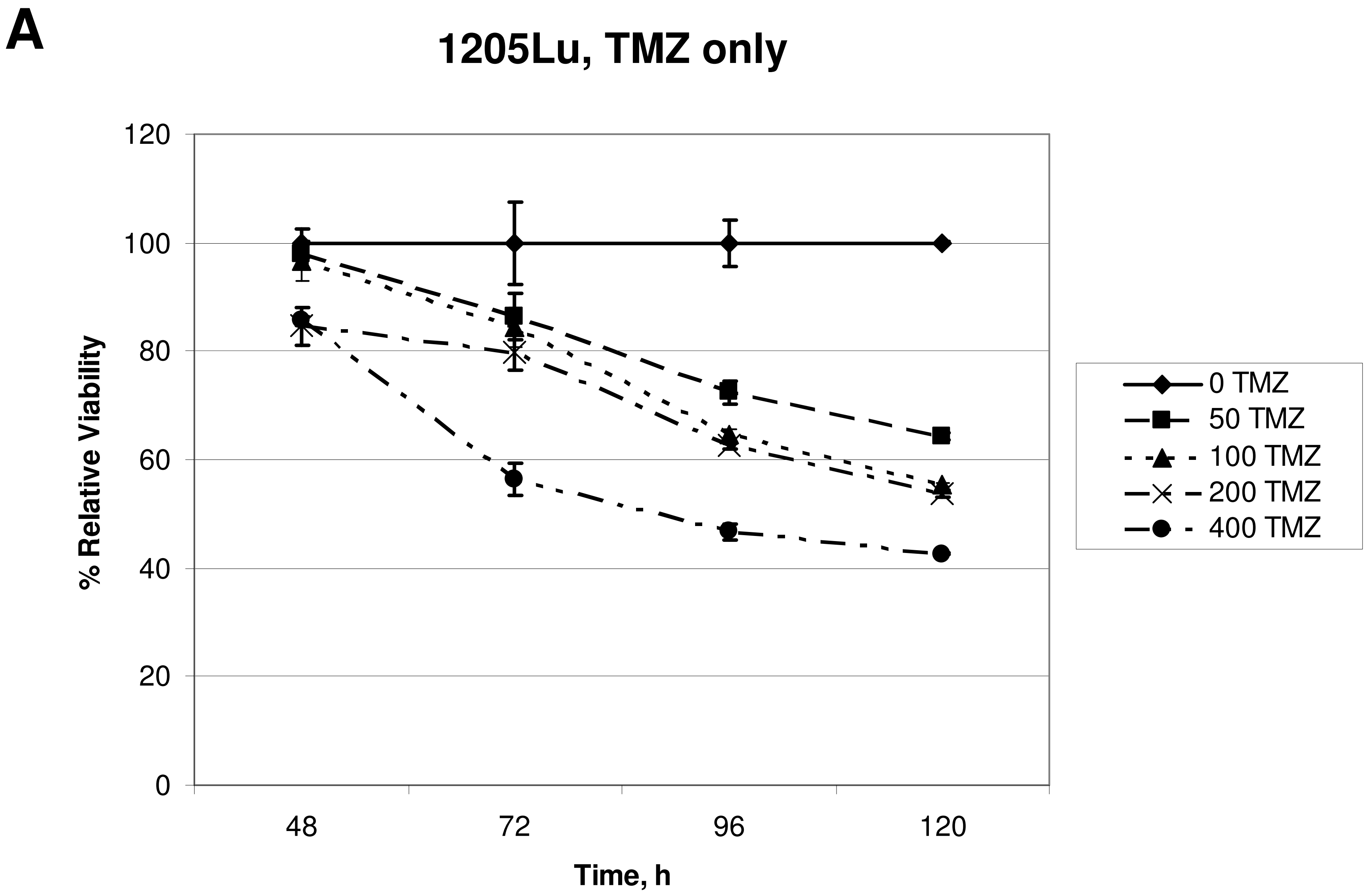

B

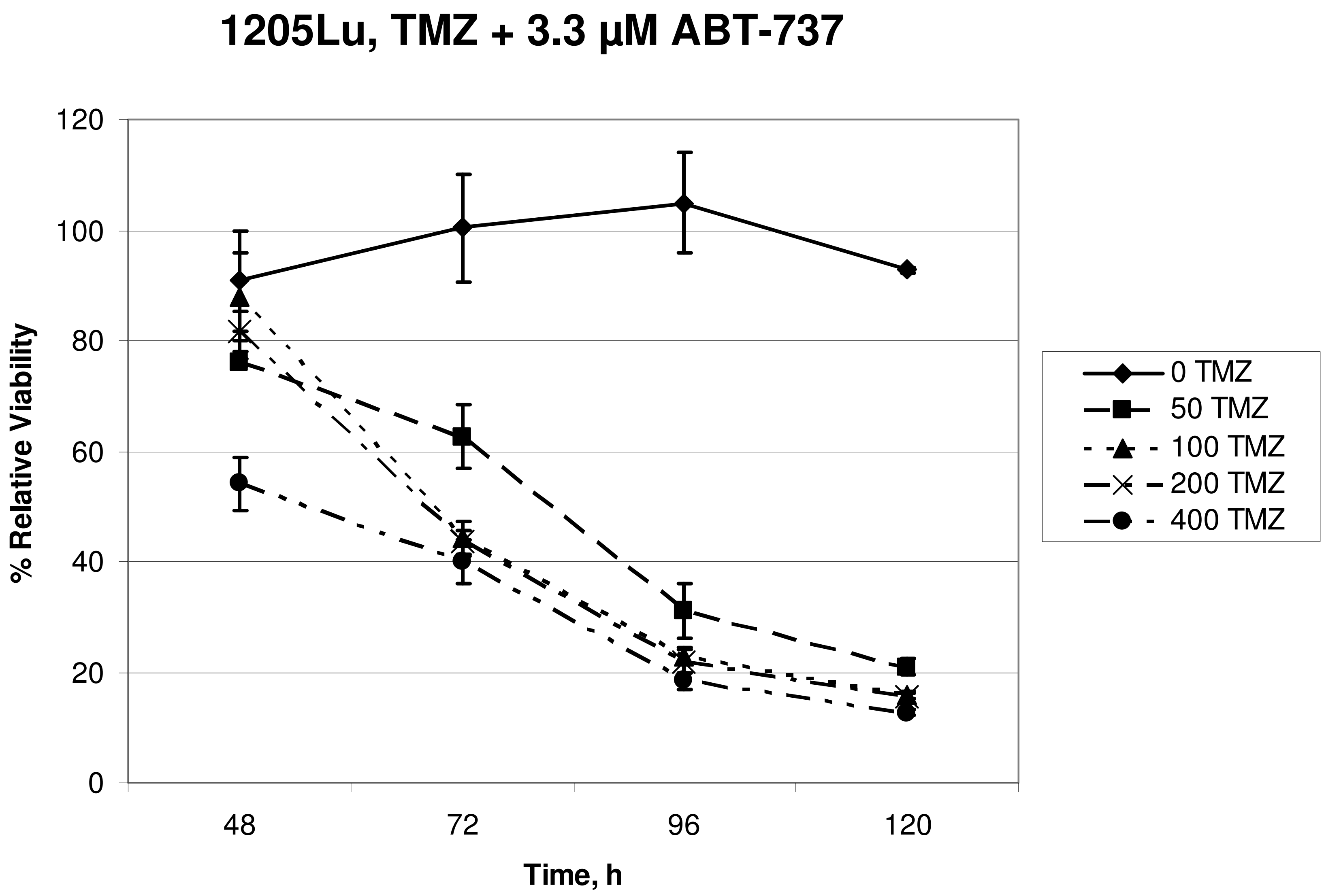
1205Lu, TMZ + 3.3 µM ABT-737
% Relative Viability
120
100
80
60
40
20
0
48
72
96
120
Time, h
0 TMZ
50 TMZ
100 TMZ
200 TMZ
400 TMZ

C

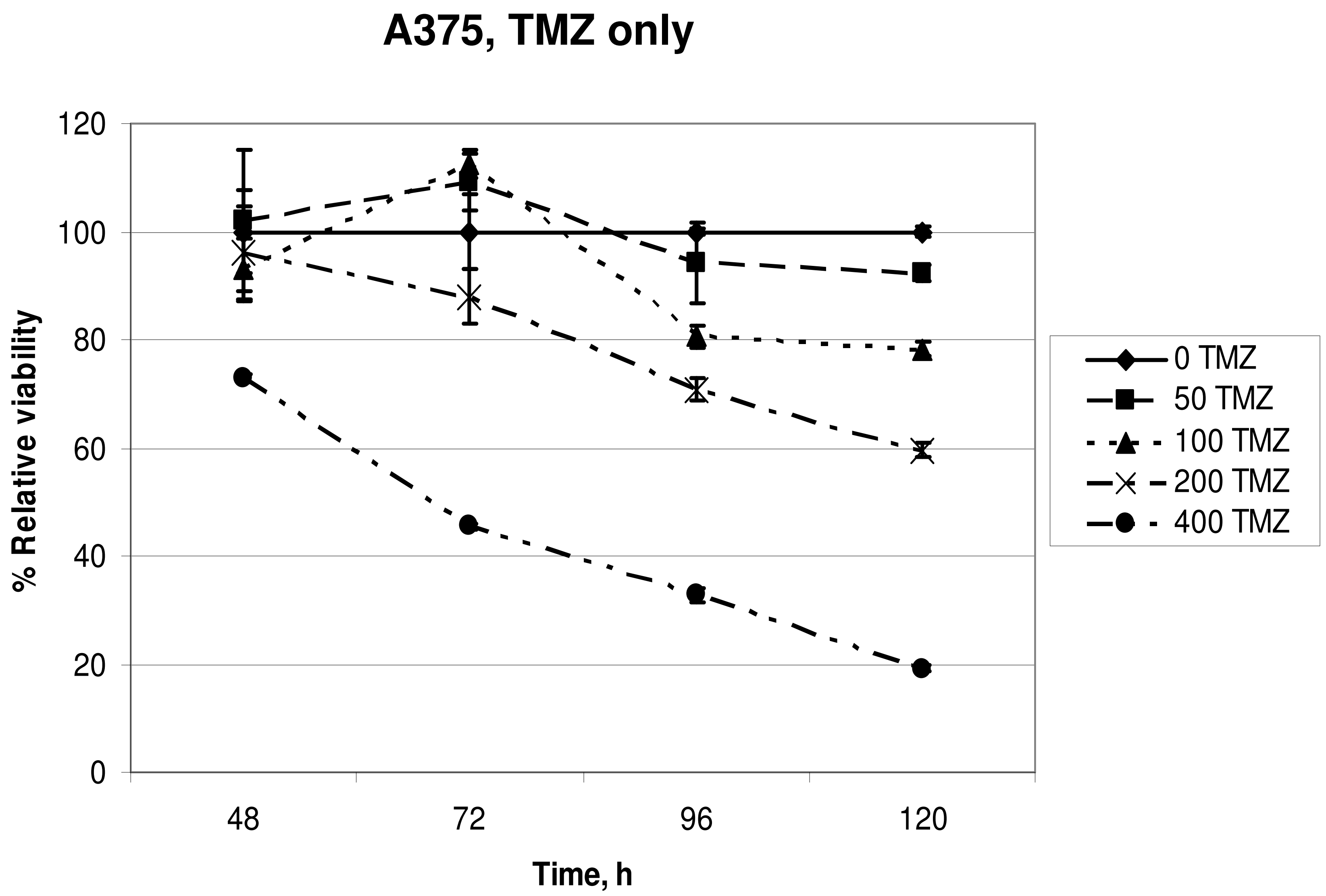

A375, TMZ only
% Relative viability
Time, h
0
20
40
60
80
100
120
48
72
96
120
0 TMZ
50 TMZ
100 TMZ
200 TMZ
400 TMZ

D

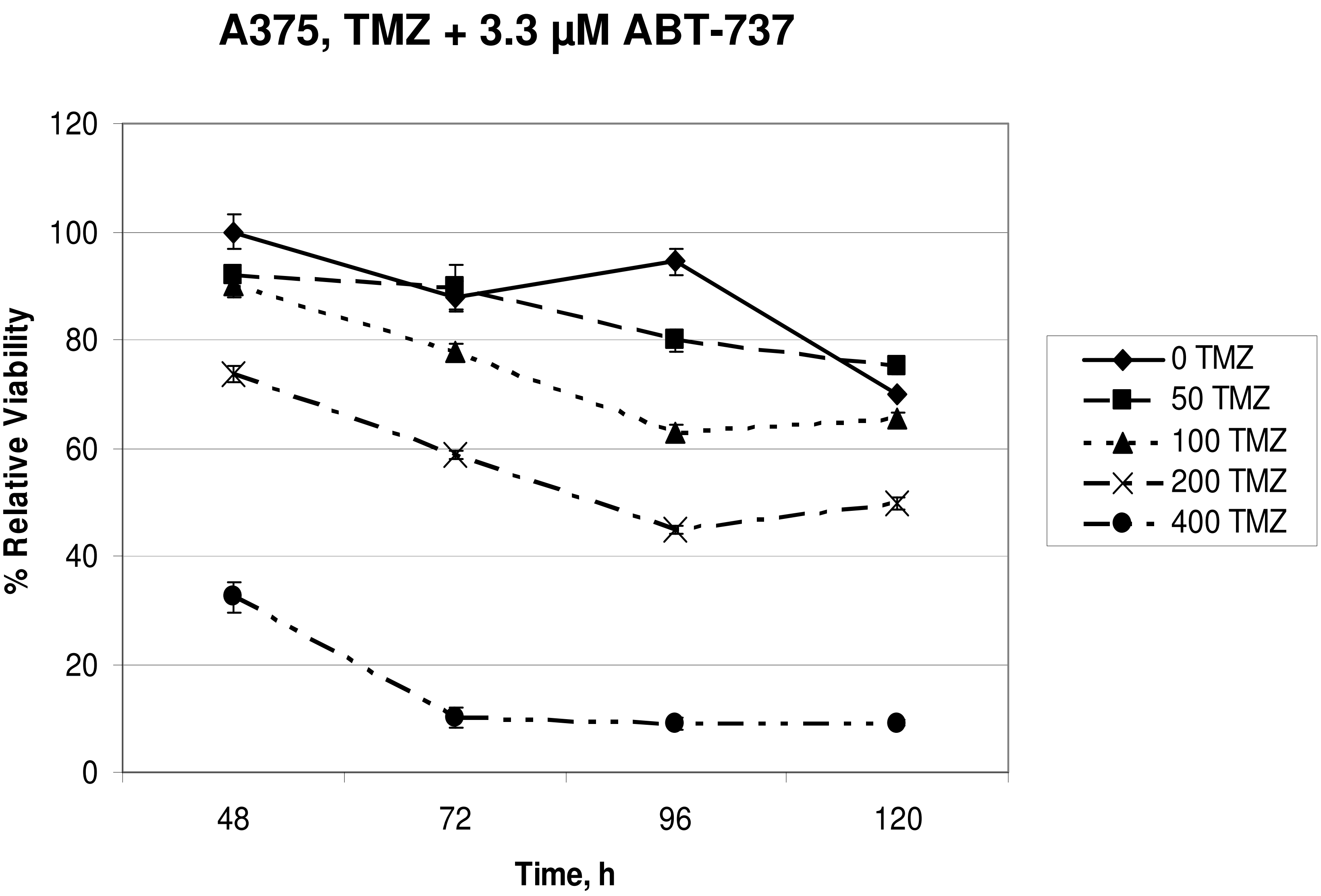

A375, TMZ + 3.3 µM ABT-737
% Relative Viability
120
100
80
60
40
20
0
48
72
96
120
Time, h
0 TMZ
50 TMZ
100 TMZ
200 TMZ
400 TMZ

Supporting Information Figure 1. Time-course experiments for cells treated with TMZ with or without ABT-737. MTS assays were performed for each 24 h time-point between 48 and 120 h for 1205Lu (**A**, **B**) and A375 (**C**, **D**) cell lines. Experiments included varying concentrations (µM) of TMZ alone (**A**, **C**) or varying concentrations of TMZ with 3.3 µM of ABT-737 (**B**, **D**). Results for each cell line are normalized to DMSO controls (0 TMZ) set at 100% for each time-point, and error bars represent +/- SE of 3 measurements.
