# Supplementary material for: The Combination of BH3-Mimetic ABT-737 with the Alkylating Agent Temozolomide Induces Strong Synergistic Killing of Melanoma Cells Independent of p53
Source: PLoS One. 2011 Aug 29;6(8):e24294. doi: 10.1371/journal.pone.0024294 (PMC3163662; doi:10.1371/journal.pone.0024294)
Supplement: Figure S2 — Fraction affected vs. Combination Index (CI) for melanoma cells treated with TMZ and ABT-737. 1205Lu cells (A) or A375 cells (B) were treated with escalating doses of TMZ (50, 100, 200, 400 µM) and ABT-737 (0.94, 1.88, 3.75, 7.5 µM) for 72 h and then subjected to MTS assays. CI plots were generated using Calcusyn software according to the Chou-Talalay method, and algebraic simulations are shown +/- estimated s.d. (PDF) [file pone.0024294.s003.pdf]

A

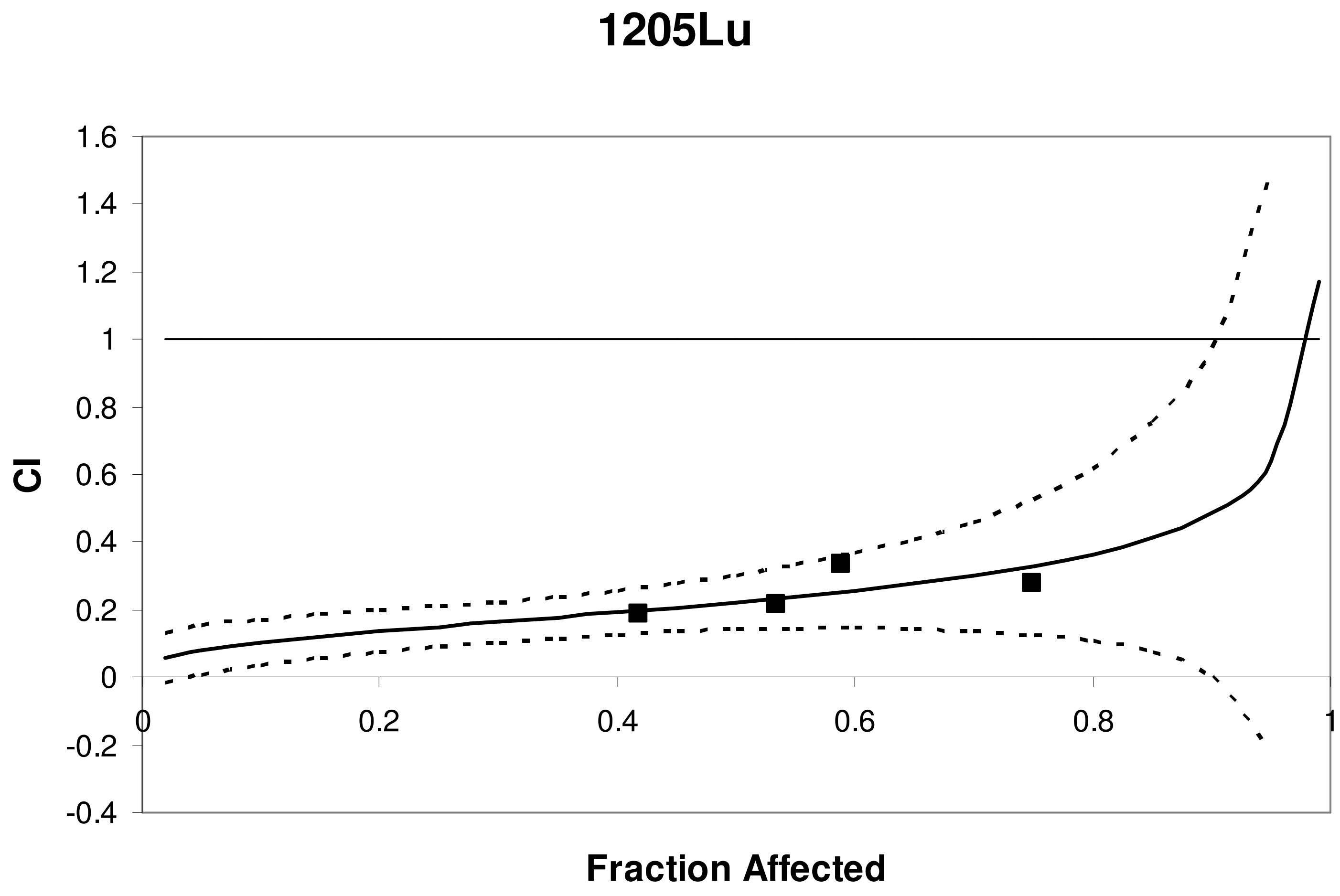

1205Lu
1.6
1.4
1.2
1
0.8
0.6
0.4
0.2
0
-0.2
-0.4
CI
0
0.2
0.4
0.6
0.8
1
Fraction Affected

B

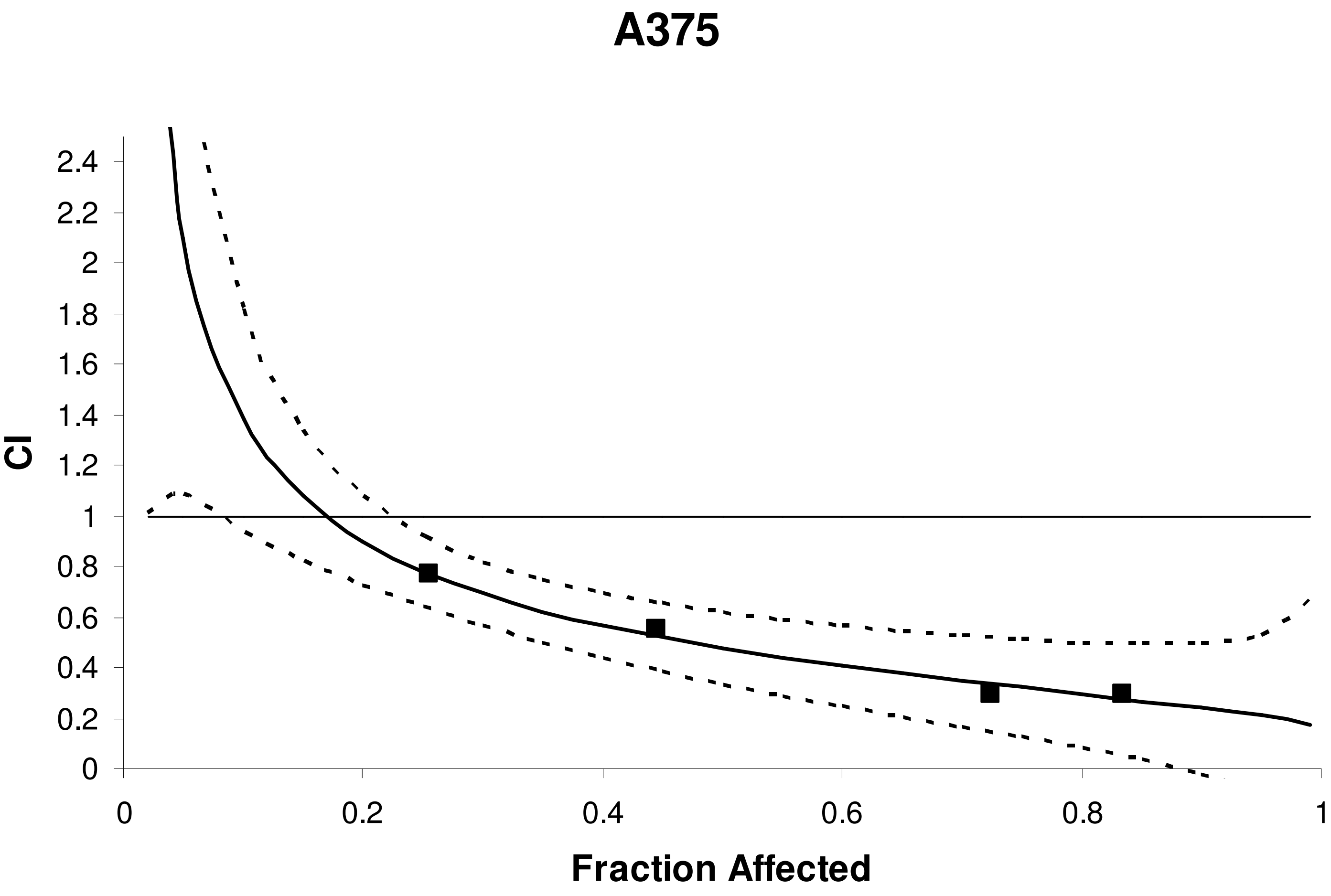

A375
CI
2.4
2.2
2
1.8
1.6
1.4
1.2
1
0.8
0.6
0.4
0.2
0
0
0.2
0.4
0.6
0.8
1
Fraction Affected

Supporting Information Figure 2. Fraction affected vs. Combination Index (CI) for melanoma cells treated with TMZ and ABT-737. 1205Lu cells (**A**) or A375 cells (**B**) were treated with escalating doses of TMZ (50, 100, 200, 400 µM) and ABT-737 (0.94, 1.88, 3.75, 7.5 µM) for 72 h and then subjected to MTS assays. CI plots were generated using Calcusyn software according to the Chou-Talalay method, and algebraic simulations are shown +/- estimated s.d.
